# The hockey-stick association of energy supply in the first 72 h of critical illness may apply only to patients with a normal body mass index: a *post-hoc* analysis of a prospective observational multicenter study

**DOI:** 10.3389/fnut.2025.1701067

**Published:** 2026-01-09

**Authors:** Youquan Wang, Yanjuan Wang, Yao Fu, Lingling Bao, Dong Zhang, Hongxiang Li

**Affiliations:** 1 Department of Critical Care Medicine, The First Hospital of Jilin University, Changchun, China; 2 Department of Neurotrauma Surgery, The First Hospital of Jilin University, Changchun, China

**Keywords:** acute gastrointestinal injury, body mass index, critical care nutrition, critically ill patients, feeding strategies, restricted cubic spline

## Abstract

**Objective:**

It is unclear whether this non-linear relationship between caloric delivery during the acute phase of critical illness and prognosis applies to patients across all body mass index (BMI) categories.

**Methods:**

This secondary analysis of a multicenter prospective observational study included patients with acute gastrointestinal injury (AGI) who were admitted to the intensive care unit (ICU) for at least 3 days. The patients were divided into two subgroups based on BMI: normal BMI and overweight (BMI: 25–30 kg/m^2^). We used univariate and multivariate Cox regression analyses to investigate the relationship between calorie delivery within the first 72 h of ICU admission and 28-day mortality and to explore whether a non-linear relationship exists between the two.

**Results:**

A total of 361 AGI patients were included in the final analysis, including 272 in the normal BMI subgroup and 89 in the overweight subgroup. In the normal BMI subgroup, Cox regression analysis revealed a significant non-linear relationship (*p* = 0.003) and association (*p* = 0.002) between daily delivered calories and 28-day mortality. Increasing the daily delivered calories from 0 to 18 kcal/kg/day was associated with decreasing mortality (hazard ratio [HR] 0.892, 95% CI: 0.816–0.975), while the daily delivered calories > 18 kcal/kg/day were associated with increasing mortality (HR: 1.116, 95% CI: 1.016–1.227). In the overweight subgroup, an increase in daily delivered calories was also associated with higher mortality (HR: 1.124, 95% CI: 1.043–1.211, *p* = 0.003). However, the overall non-linear relationship between daily delivered calories and mortality disappeared (*p* = 0.466). Notably, increasing the daily delivered calories from 15 to 25 kcal/kg/day was associated with increased mortality (HR: 1.160, 95% CI: 1.030–1.306). After adjusting for potential confounders through a multivariable Cox regression analysis, the results remained robust.

**Conclusion:**

The association between daily delivered calories within the first 72 h of ICU admission and 28-day mortality in critically ill patients showed a hockey-stick association, which was observed only in patients with a BMI of 18.5–24.9 kg/m^2^. However, no similar relationship was found in patients with a BMI of 25–30 kg/m^2^. These findings should be interpreted as exploratory and hypothesis-generating, and further studies are needed to confirm and extend these observations.

## 1 Introduction

Malnutrition is prevalent among intensive care unit (ICU) patients. This is frequently attributed to inadequate nutrition prior to ICU admission and the profound hyper-catabolic state induced by conditions such as sepsis and trauma, as outlined in the European Society of Intensive Care Medicine’s (ESICM) guidelines (1). This leads to immune impairment, increased infection susceptibility, compromised healing, and ultimately higher mortality rates in critically ill patients (2, 3).

Nutritional support plays a crucial role in managing critically ill patients and can be administered through enteral feeding or parenteral feeding. Parenteral nutrition (PN) is used when enteral nutrition (EN) is inadequate or not feasible. However, the optimal dosage of nutritional support during the early phase of critical illness remains controversial (4, 5). Zusman et al. first revealed the “U-shaped curve” of calorie supply in critically ill patients through retrospective studies (6), indicating that both insufficient and excessive calorie supply are detrimental. The most beneficial energy supply may be one that reaches approximately 70% of the resting energy expenditure on average per day. Overfeeding can impose a digestive burden, suppress beneficial metabolic processes such as autophagy and ketosis, and may also lead to “hibernating mitochondria to die of overwork” (7, 8, 9). This theory explains the existence of the “U-shaped curve,” showing that higher nutritional supply is not always better. The subsequent PROTINVENT study also suggests that protein supply, such as calories, may follow the non-linear relationship, with both excessive and insufficient protein intake being associated with poor prognosis in critically ill patients (10). Gradual feeding may be optimal, aligning with the feeding strategies recommended by the European Society for Clinical Nutrition and Metabolism (ESPEN) guidelines (11).

However, the harm caused by inadequate feeding may vary for each patient. During the acute phase of critical illness, the body is in a state of high catabolism (9, 11), and patients with poor nutritional reserves may be more vulnerable to the consequences of underfeeding and thus warrant closer nutritional attention, whereas the harm of inadequate feeding may be less significant for those with better nutritional reserves (12, 13). Body mass index (BMI) may, to some extent, represent nutritional reserves (14). Therefore, we conducted a secondary analysis of a prospective observational study recruiting critically ill patients with acute gastrointestinal injury (AGI), which refers to the dysfunction of the gastrointestinal tract due to their acute illness (1). We hypothesized that the non-linear relationship of energy supply applies only to patients with a normal BMI, but not to those with a BMI greater than 25 kg/m^2^.

## 2 Methods

### 2.1 Participants

We used data from a prospective, observational, nationwide study conducted between September 2014 and December 2014 in China, which originally aimed to investigate the association between nutrition support and prognosis in critically ill patients with AGI (15). The names and geographical locations of the 12 teaching hospitals are in the Supplementary Document. The project protocols followed the Strengthening the Reporting of Observational Studies in Epidemiology (STROBE) guidelines (16). Patients meeting the ESICM’s definition and grading system for AGI were eligible for inclusion if they also received nutrition support within 72 h of admission (17). Individuals with severe cardiovascsular disease, post-cardiac arrest, chronic end-stage organ failure, malignancy, Crohn’s disease, ulcerative colitis, or short bowel syndrome, as well as those who died within 72 h or were hospitalized for less than 72 h before AGI was diagnosed, were excluded from this study. This study followed the feeding strategies advocated by the 2012 ESICM guidelines (caloric targets: 25–30 kcal/kg/day). The study was approved by the Ethics Committee of the First Hospital of Jilin University in December 2012 (No. 2012–088). The study was conducted in 2014 without clinical registration, and the original results have been previously published (18).

All patients from the original prospective observational study were included in this analysis. The exclusion criteria were as follows: missing nutrition data or AGI grade, treatment abandonment, hospitalization duration of less than 72 h, and patients with underweight (BMI < 18.5 kg/m^2^) or obesity (BMI ≥ 30 kg/m^2^). Patients at these BMI extremes were excluded because their numbers in this cohort were very small, which precluded reliable subgroup analyses and would have yielded highly unstable and potentially misleading estimates.

### 2.2 Data collection

The enrolled patients were categorized into subgroups based on the BMI cutoff values from previous studies (19, 20), with one subgroup being the normal BMI (18.5 ≤ BMI < 25 kg/m^2^) and the other subgroup being the overweight (BMI: 25–30 kg/m^2^). For individuals with a normal BMI, actual body weight (ABW) was used to determine energy requirements. For those with a BMI of 25–30 kg/m^2^, the reference (adjusted) body weight was replaced with the ideal body weight (IBW). The IBW was calculated by subtracting 100 (for male individuals) or 106 (for female individuals) from 0.9 multiplied by the height in centimeters. When calculating energy requirements, we used adjusted body weight, defined as ideal body weight plus 20% of the excess weight (i.e., the difference between actual body weight and ideal body weight), in accordance with the recommendation of the 2023 ESPEN guidelines (11).

Detailed information on nutritional supply via EN and PN during the first 72 h after ICU admission was collected from the bedside nursing flow sheets. For each 24-h period, the actually administered volume of every EN and PN formula was recorded. Total daily energy intake (kcal/day) was calculated by multiplying the administered volume of each product by its labeled energy density (kcal/mL) and summing the energy provided by EN and PN. The collected data also included general demographics, primary diagnosis, Acute Physiology and Chronic Health Evaluation (APACHE II) score, AGI grade, and 28-day mortality (telephone follow-up or investigation from the public security system). These parameters were used for comprehensive analysis and evaluation. The data were thoroughly screened for missing information and any implausible or outlier values.

### 2.3 Statistical analyses

Continuous variables were expressed as means and standard deviations for normally distributed variables using the *t*-test. The median and interquartile range (IQR) were reported for non-normally distributed variables, and the Kruskal–Wallis test was used for evaluation. Categorical variables were compared using the chi-squared test.

We performed a subgroup analysis based on whether the patients had a BMI of 25–30 kg/m^2^. Initially, an unadjusted Cox proportional hazards model for 28-day mortality was used, with the daily delivered calories entered as a continuous variable. Given the potential non-linearity of this relationship, daily calorie delivery was modeled as a continuous variable using restricted cubic splines with three pre-specified knots. Given the moderate overall sample size (<400 patients) and approximately 200 patients in each BMI subgroup, a parsimonious 3-knot specification was chosen to balance flexibility and model stability; knots were placed at the 10th, 50th, and 90th percentiles of daily energy delivery.

Subsequently, the Cox regression results were adjusted for potential risk factors affecting mortality in different subgroups to explore the association and non-linear relationship between daily delivered calories and the adjusted hazard ratio (HR) for 28-day mortality. For each BMI subgroup, we considered three potential covariates—APACHE II score, AGI grade, and mechanical ventilation—as risk factors for 28-day mortality. These variables were then entered into the Cox regression analyses. Variables showing a significant univariate association with 28-day mortality and without multicollinearity (assessed via variance inflation factors and R^2^) were included in the final adjusted model. This ensured subgroup-specific confounder control. Non-linearity was assessed using an analysis of variance (ANOVA) to compare the spline model to the linear term model.

The proportional hazards assumption was evaluated for all Cox models using visual inspection of log–minus–log survival plots and using tests based on scaled Schoenfeld residuals. Model diagnostics further included the inspection of martingale and deviance residuals, as well as DFBETAs (difference-in-betas) influence statistics for the spline coefficients, to assess overall model fit and identify influential observations. A *p*-value of < 0.05 was considered statistically significant. The statistical analysis was conducted using SPSS for Mac version 26 (SPSS Inc., Chicago, IL, USA) and Rv4.3.1 (R Foundation for Statistical Computing, Vienna, Austria) using RStudio v1.0.136 (RStudio Inc., Boston, MA, USA).

## 3 Results

### 3.1 Baseline characteristics

A total of 443 patients were initially enrolled in the original study based on the inclusion criteria. Among them, 64 individuals were excluded due to missing nutritional data or AGI grade, treatment abandonment, or a hospitalization duration of less than 72 h, while 379 patients were retained for the final analysis of the original study. For the current secondary analysis, we further excluded patients who were underweight (BMI < 18.5 kg/m^2^) (10 individuals) and obese (BMI ≥ 30 kg/m^2^) (8 individuals) due to the potential impact of small sample sizes on the reliability of the outcomes. Consequently, 361 patients were included in the final analysis.

**Figure 1:**
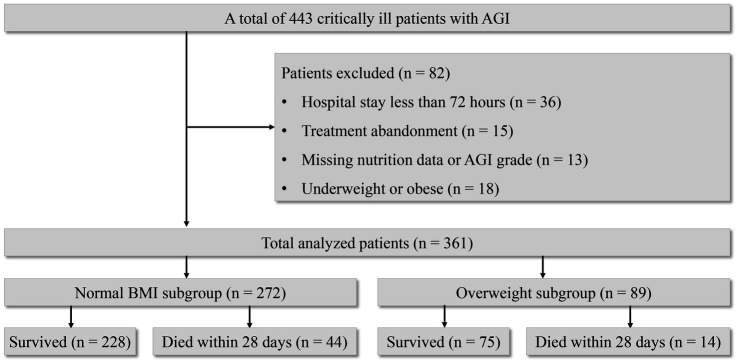
Patient inclusion flowchart.

**Figure 2:**
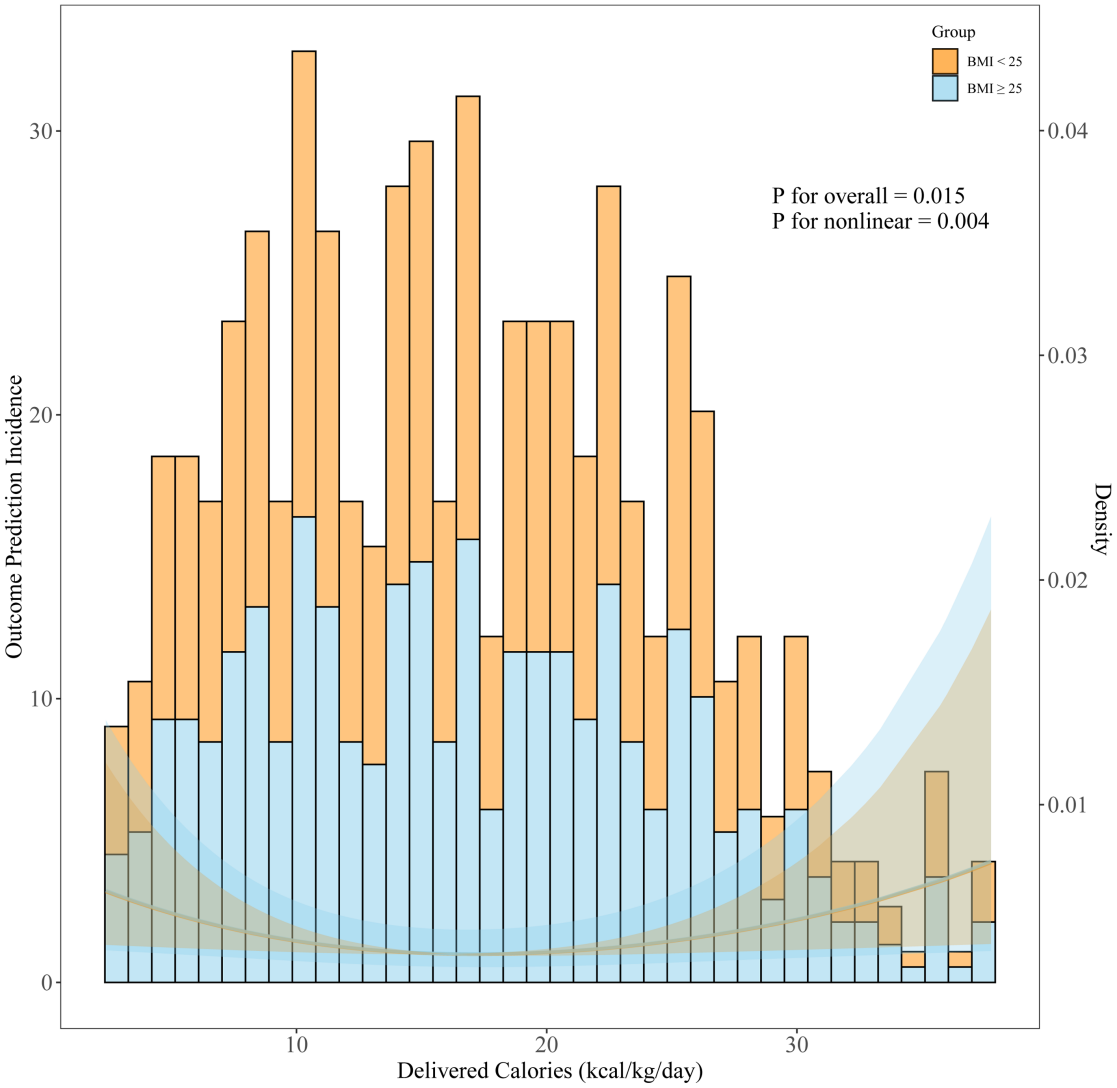
Association between daily delivered calories and 28-day mortality in all patients, modeled using an unadjusted Cox proportional hazards regression analysis with restricted cubic splines (three knots). The solid curve corresponds to the left Y-axis (risk of 28-day mortality, %), showing a non-linear relationship between calorie delivery (kcal/kg/day) and mortality. The histogram corresponds to the right Y-axis, representing the proportion of patients in each BMI subgroup (BMI: 18.5–24.9 vs. 25–30 kg/m^2^), displayed on a 0–1 scale (i.e., the decimal form of %). *p*-values indicate the overall and non-linear significance of the spline terms, based on Wald tests.

All patients were divided into two subgroups according to their BMIs, with 272 patients (75.3%) in the normal BMI subgroup and 89 patients (24.7%) in the overweight subgroup (Figure 1 and Table 1). The daily average of delivered calories over the first 72 h in the normal BMI subgroup and the overweight subgroup is shown in Figure 2.

**Table 1:** Clinical and demographic data for development and validation cohort.

Variables	Normal BMI subgroup				Overweight subgroup			
	Total (*n =* 272)	Survived (*n =* 228)	Died within 28 days (*n =* 44)	*p-*value	Total (*n =* 89)	Survived (*n =* 75)	Died within 28 days (*n =* 14)	*P-*value
Age, y	61.8 ± 18.7	61.5 ± 18.0	63.8 ± 22.1	0.443	57.6 ± 18.1	56.8 ± 17.5	62.1 ± 20.6	0.309
Sex, *n* (%)								
Male	183 (67.3)	154 (67.5)	29 (65.9)	0.832	59 (66.3)	50 (66.7)	9 (64.3)	0.863
Female	89 (32.7)	74 (32.5)	15 (34.1)		30 (33.7)	25 (33.3)	5 (35.7)	
BMI	22.6 ± 1.5	22.7 ± 1.5	22.2 ± 1.7	0.098	26.7 ± 1.2	26.5 ± 1.1	27.3 ± 1.5	0.078
Primary diagnosis, *n* (%)				0.514				0.101
Neurological	55 (20.2)	47 (20.6)	8 (18.2)		16 (18.0)	10 (13.3)	6 (42.9)	
Circulatory	18 (6.6)	16 (7.0)	2 (4.5)		5 (5.6)	4 (5.3)	1 (7.1)	
Respiratory	116 (42.6)	99 (43.4)	17 (38.6)		46 (51.7)	41 (54.7)	5 (35.7)	
Polytrauma	18 (6.6)	16 (7.0)	2 (4.5)		5 (5.6)	5 (6.7)	0 (0)	
Others	65 (23.9)	50 (21.9)	15 (34.1)		17 (19.1)	15 (20.0)	2 (14.3)	
Severity of illness^a^								
APACHE II score	16 (12–22)	15 (11–20)	22 (18–26)	< 0.001	16 (12–21)	15 (10–18)	22 (17–25)	0.005
SOFA score	6 (3–8)	5 (3–8)	8 (5–10)	< 0.001	6 (3–10)	5 (3–7)	8 (4–12)	0.009
mNUTRIC	5 (4–6)	5 (3–6)	6 (5–7)	< 0.001	4 (3–6)	4 (3–6)	5 (4–7)	0.122
AGI grade, *n* (%)				0.003				0.831
I	102 (37.5)	91 (39.9)	11 (25.0)		34 (38.2)	30 (40.0)	4 (28.6)	
II	129 (47.4)	110 (48.2)	19 (43.2)		36 (40.4)	32 (42.7)	4 (28.6)	
III	28 (10.3)	20 (8.8)	8 (18.2)		16 (18.0)	13 (17.3)	3 (21.4)	
IV	13 (4.8)	7 (3.1)	6 (13.6)		3 (3.4)	0 (0)	3 (21.4)	
Underlying disease, *n* (%)								
Hypertension	137 (50.4)	118 (51.8)	19 (43.2)	0.298	57 (64.0)	48 (64.0)	9 (64.3)	0.984
Diabetes	86 (31.7)	72 (31.7)	14 (31.8)	0.990	26 (29.2)	21 (28.0)	5 (35.7)	0.560
Primary AGI, *n* (%)	143 (52.6)	119 (52.2)	24 (54.5)	0.775	35 (39.3)	28 (37.3)	7 (50.0)	0.373
Mechanical ventilation, *n* (%)	181 (66.5)	144 (63.2)	37 (84.1)	0.007	56 (62.9)	45 (60.0)	11 (78.6)	0.187
Surgical admission, *n* (%)	129 (47.4)	113 (49.6)	16 (36.4)	0.108	35 (39.3)	27 (36.0)	8 (57.1)	0.137
Emergency admission	119	104	15	0.213	29	21	8	0.068
Elective admission	10	9	1	0.918	6	6	0	0.584
Sepsis, *n* (%)	115 (42.3)	97 (42.5)	18 (40.9)	0.869	37 (41.6)	29 (38.7)	8 (57.1)	0.198
EN start time, h	48 (24–72)	48 (24–72)	48 (24–72)	0.795	48 (24–72)	48 (24–72)	48 (24–54)	0.765
EN initial rate, ml/h	20 (20–20)	20 (20–20)	20 (20–20)	0.946	20 (20–20)	20 (20–20)	20 (17.5–20)	0.128
Daily delivered calories ^b^, kcal/kg/day	16.7 (10.2–22.9)	16.9 (11.1–22.7)	11.1 (8.3–25.9)	0.046	15 (9.3–22.3)	14.5 (8.8–18.8)	24.1 (18.7–25.5)	0.002
Energy intake of EN in 72 h ^b^, median [IQR], kcal/kg/day	1.7 (0–7.7)	1.5 (0–7.8)	2.8 (0–7.4)	0.641	0 (0–4.8)	0 (0–4.8)	0 (2–4.1)	0.872
Energy intake of PN in 72 h ^b^, median [IQR], kcal/kg/day	11.4 (5.6–19.3)	11.8 (6.0–19.2)	8.3 (2.0–21.8)	0.272	11.1 (5.6–18.6)	10.0 (5.6–15.4)	22.9 (16.6–25.1)	0.002
Tube feeding route, n (%)				0.932				0.431
Prepyloric	236 (86.8)	198 (86.8)	38 (86.4)		76 (85.4)	65 (86.7)	11 (78.6)	
Postpyloric	36 (13.2)	30 (13.2)	6 (13.2)		13 (14.6)	10 (13.3)	3 (21.4)	
LOS in ICU, day	11 (8–21)	11.5 (8–22)	10 (6–16)	0.124	11 (8–21)	11 (7–20)	17 (9–26)	0.099
Hospital-acquired infection	101 (37.3)	85 (37.4)	16 (36.4)	0.892	37 (41.6)	28 (37.3)	9 (64.3)	0.060

BMI, body mass index; APACHE II, Acute Physiology and Chronic Health Evaluation II; SOFA, sequential organ failure assessment; AGI, acute gastrointestinal injury; EN, enteral nutrition; IQR, interquartile range. Data are presented as mean ± standard deviation, median (IQR), or n (%). ^a^Indicated within 24 h of admission to the ICU; ^b^ daily average of delivered calories over the first 72 h.

### 3.2 The association between the daily delivered calories and 28-day mortality

#### 3.2.1 Normal BMI subgroup

**Figure 3:**
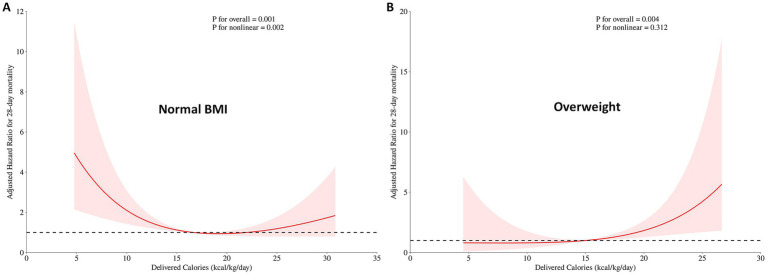
Association between daily delivered calories and the HR for 28-day mortality in critically ill patients stratified by BMI. **(A)** In patients with a normal BMI, a significant non-linear (hockey stick) relationship was observed between calorie delivery and the HR for 28-day mortality, with the lowest HR occurring at approximately 18 kcal/kg/day. **(B)** In patients who were overweight, no evidence of the hockey-stick association was found. The red line represents the estimated HR derived from restricted cubic spline regression, and the shaded area indicates the 95% confidence interval. *p-values* represent the significance of the overall association and the test for non-linearity.

When the daily delivered calories were examined as a continuous variable in relation to 28-day mortality, a significantly non-linear pattern (hockey-stick association) was demonstrated in the normal BMI subgroup (*p* = 0.003) (Figure 3A), with a significant association with 28-day mortality (*p* = 0.002). The lowest 28-day mortality was observed at approximately 18 kcal/kg/day, representing the nadir of the hockey-stick association. Increasing the daily delivered calories from 0 to 18 kcal/kg/day (approximately 70% of the nutritional target) was associated with a decreased risk of mortality (hazard ratio [HR] 0.892, 95% CI: 0.816–0.975), while the daily delivered calories > 18 kcal/kg/day were associated with increased mortality (HR: 1.116, 95% CI: 1.016–1.227).

**Figure 4:**
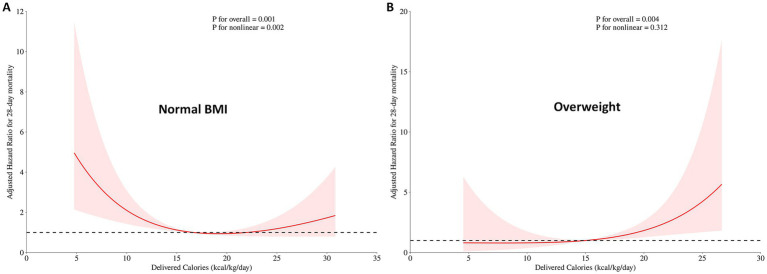
Association between daily delivered calories and adjusted HR for 28-day mortality in critically ill patients stratified by BMI. **(A)** In patients with a normal BMI, the hockey-stick association was observed after adjusting for APACHE II score, mechanical ventilation, and daily delivered calories. **(B)** In patients who were overweight, no evidence of the hockey-stick association was observed after adjusting for the APACHE I score, AGI grade, and daily delivered calories. The red line represents the adjusted HR estimated using restricted cubic spline Cox regression analysis, and the shaded area indicates the 95% confidence interval. *p*-*values* represent the significance of the overall association and the test for non-linearity.

In the normal BMI subgroup, the final Cox model included APACHE II score, mechanical ventilation, and daily delivered calories to assess the non-linear relationship and the robustness of the association between daily delivered calories and the HR for 28-day mortality (Table 2). We found that, after adjusting for covariates, the non-linear relationship (*p* = 0.002) and association (*p* = 0.001) between daily delivered calories and the HR for 28-day mortality remained robust (Figure 4A). The proportional hazards assumption was not violated (global test based on Schoenfeld residuals: *χ*^2^ = 1.52, df = 2, *p* = 0.47).

**Table 2:** Multivariate predictors of 28-day mortality in the normal BMI subgroup.

Variable	*OR*	(95%CI)	*P*-value
APACHE II score	1.104	(1.056–1.154)	< 0.001
AGI grade	1.279	(0.919–1.779)	0.145
Mechanical ventilation	2.599	(1.146–5.893)	0.022
Daily delivered calories	0.961	(0.923–1.001)	0.057

BMI, body mass index; APACHE, Acute Physiology and Chronic Health Evaluation; AGI, acute gastrointestinal injury.

Model diagnostics are shown in Supplementary Figures S1A–C. Martingale residuals plotted against daily energy delivery (Supplementary Figure S1A) did not exhibit a strong systematic pattern, indicating that the restricted cubic spline specification adequately captured the functional form of the exposure. Deviance residuals vs. the linear predictor (Supplementary Figure S1B) showed no pronounced trends or clustering suggestive of a major lack of fit. Influence diagnostics based on DFBETAs for the spline coefficients (Supplementary Figure S1C) demonstrated uniformly small values, indicating that no single observation exerted undue influence on the estimated dose–response relationship.

#### 3.2.2 Overweight subgroup

In the overweight subgroup, an increase in daily delivered calories was associated with an increased 28-day mortality (HR: 1.124, 95% CI: 1.043–1.211, *p* = 0.003). A similar non-linear relationship was not observed between daily delivered calories and 28-day mortality (*p* = 0.466). Daily delivered calories > 15 kcal/kg/day (approximately 60% of the nutritional target) were associated with an HR > 1. Increasing the average of daily delivered calories from 15 to 25 kcal/kg/day within the first 72 h was associated with increased mortality (HR: 1.160, 95% CI: 1.030–1.306) (Figure 3B).

In the overweight subgroup, the final Cox model included APACHE II score, AGI grade, and daily delivered calories (Table 3). We found that, after adjusting for covariates, the association between daily delivered calories and the HR for 28-day mortality (*p* = 0.004) remained robust, and no non-linear relationship was found between the two (*p* = 0.312) (Figure 4B). The proportional hazards assumption was not violated (global test based on Schoenfeld residuals: *χ*^2^ = 4.48, df = 2, *p* = 0.11).

**Table 3:** Multivariate predictors of 28-day mortality in the overweight subgroup.

Variable	*OR*	(95% CI)	*P*-value
APACHE II score	1.108	(1.033–1.189)	0.004
AGI grade	2.379	(1.250–4.528)	0.008
Daily delivered calories	1.161	(1.060–1.272)	0.001

APACHE, Acute Physiology and Chronic Health Evaluation; AGI, acute gastrointestinal injury.

Model diagnostics are shown in Supplementary Figures S2A–C. Martingale residuals plotted against daily energy delivery (Supplementary Figure S2A) were mostly centered around zero, with only mild curvature at higher intake levels, suggesting that the restricted cubic spline specification adequately captured the functional form of the exposure. Deviance residuals vs. the linear predictor (Supplementary Figure S2B) did not exhibit pronounced trends or clustering indicative of a major lack of fit. Influence diagnostics based on DFBETAs for the spline coefficients (Supplementary Figure S2C) showed uniformly small values (all far below the conventional 2/√n threshold), indicating that no single observation exerted undue influence on the estimated association.

In the overweight subgroup, the relationship with 28-day mortality was not strictly linear, as an increased risk was observed only when daily calorie delivery exceeded 15 kcal/kg/day (Supplementary Table S1).

## 4 Discussion

In the *post-hoc* analysis of this prospective multicenter observational study, we found a significant non-linear association between the average of daily delivered calories in the first 72 h after ICU admission and 28-day mortality in the normal BMI subgroup, revealing the “hockey-stick association” for calorie supply. As daily delivered calories increased over the first 72 h, mortality decreased when the intake increased from 0 to approximately 18 kcal/kg/day; however, increases above this point were associated with higher mortality. In the overweight subgroup, higher daily delivered calories were associated with increased 28-day mortality, and no similar non-linear relationship between daily delivered calories and mortality was observed. These patterns remained essentially unchanged after multivariable adjustment for relevant covariates. In both BMI subgroups, the proportional hazards assumption was not violated in the spline-based Cox models, and brief model diagnostics (residual plots and influence analyses) did not reveal a major lack of fit or highly influential observations, suggesting that the estimated associations are relatively stable, albeit exploratory.

In patients with a normal BMI, the non-linear association between early energy supply and mortality was clearly asymmetric. The slope of the curve was much steeper at the lower end of calorie intake, indicating that energy deficit was associated with a higher risk of mortality, whereas at higher intake levels, the curve continued to rise gradually, without a similarly steep increase in mortality. This pattern suggests that insufficient energy delivery may confer disproportionately greater harm than modest energy excess in this population. For this reason, we described the observed relationship as a “hockey stick” rather than a traditional “U-shaped” curve, which typically implies a more symmetric and similarly steep increase in risk at both low and high levels of exposure.

Zusman et al. were the first to observe the non-linear relationship of calorie delivery, suggesting that an average daily calorie intake of approximately 70% may be optimal, while both excessive and insufficient calorie delivery could be harmful (6). This intriguing phenomenon can be explained by the risks of malnutrition and overfeeding. During the acute phase of critical illness, mitochondrial dysfunction occurs (9, 21), and overfeeding increases the metabolic burden while inhibiting beneficial metabolic processes such as ketosis and autophagy (3, 7, 22, 23). Therefore, in both BMI subgroups, the trend toward higher mortality with acute-phase overfeeding was similar and largely expected. Surprisingly, however, was the strikingly different association between low caloric intake and mortality in the two subgroups. Compared with overweight patients, those with a normal BMI are likely to have relatively limited reserves of muscle and adipose tissue. When exposed to the metabolic stress of critical illness, these patients may rely more heavily on exogenous energy provision, such that energy deficit or underfeeding becomes a biologically plausible explanation for their worse prognosis. For overweight or obese patients, endogenous energy reserves are relatively larger, allowing them to better cope with insufficient nutritional supply. This theory seems to resemble the “obesity paradox (24, 25).”

Our research findings also support this hypothesis. We validated the hockey-stick association of calorie delivery in the normal BMI subgroup, which is similar to the results of Zusman et al. (6) While their study focused on daily calorie delivery during the ICU stay, our study concentrated on daily calorie delivery during the first 72 h after ICU admission, as we believe that early nutritional support during the acute phase is more crucial and has a greater impact on prognosis. Although several meta-analyses did not demonstrate an effect of calorie intake on mortality across different BMI subgroups (26, 27, 28), it should be noted that average intake over the whole ICU stay is fundamentally different from calorie delivery in the very early acute phase. Another aim of our study was to question whether the hockey-stick association in calorie delivery also exists for patients with a BMI of 25–30 kg/m^2^. Higher nutritional delivery in the overweight subgroup appeared to act as a “sweet burden,” potentially contributing to higher mortality (24, 29). Although the results remained consistent with the unadjusted analysis, further high-quality studies are needed to validate these findings.

This study has several key strengths: 1. It is the first study to propose that the hockey-stick association between calorie delivery and mortality does not apply to all critically ill patients. We validated our initial hypothesis that the hockey-stick association only applies to patients with a normal BMI, which represents the most innovative aspect of this study; 2. the results of this study remained consistent after adjusting for key confounding factors, suggesting a potentially reliable association. However, as only a limited number of covariates were included in the multivariable model, the possibility of residual confounding cannot be excluded; and 3. this exploratory study may offer preliminary insights that could inform future research on personalized nutrition strategies for critically ill patients, particularly in understanding the potential hockey-stick association between nutritional delivery and outcomes.

There are some limitations to this study. First, although this is a post-hoc analysis of a prospective, multicenter observational study involving 12 teaching hospitals in China, the sample size remains relatively small, which may affect the robustness of some outcomes. In addition, continuous variables were assumed to have linear effects, which may overlook potential non-linear relationships. Moreover, our analysis may be affected by confounding for indication and survival bias, which are inherent limitations of observational studies. Therefore, our findings should be regarded as exploratory, and high-quality prospective randomized controlled trials are required to validate these observations. Second, only AGI patients were included in this study, not all critically ill patients. Although the exposure variable in this study was the total caloric intake from both EN and PN—which may reduce the potential confounding effect of gastrointestinal dysfunction compared with EN alone—it remains unclear whether the findings can be generalized to all critically ill patients. Further validation is needed to determine if the findings apply to all critically ill patients. Third, the calculation of reference body weight for overweight individuals has not been definitively established. In this study, we adopted the method recommended by the guideline, but further research is needed to determine its broader applicability (30). Due to the limited number of cases, this study did not include patients with BMIs greater than 30 kg/m^2^ or less than 18.5 kg /m^2^. While categorizing patients based on whether their BMI exceeds 25 kg/m^2^ could offer more generalizable findings, the very small number of patients in the underweight and obese categories increased the potential unreliability of the outcomes. Theoretically, underweight patients may be more dependent on nutrition, and obese patients may share similar characteristics with overweight individuals. However, this remains speculative, and further research with larger datasets is required to validate this hypothesis. Additionally, the BMI does not fully reflect body composition distribution, and further research is needed to explore differing values in nutritional support (30). Fourth, there is significant heterogeneity in critically ill patients, and the associations between various factors and the presence of nutritional support remain unclear (31). These uncertainties may have an impact on the findings of this study. Fifth, we did not measure energy expenditure using indirect calorimetry and therefore could not assess the adequacy of energy delivery relative to patients’ true metabolic needs. In addition, detailed protein intake data were not collected or analyzed, and non-nutritional sources of calories (e.g., dextrose-containing fluids, propofol) were not systematically quantified. These limitations may have led to some misclassification of actual energy and protein provision and could have affected the observed associations between calorie intake and outcomes. Sixth, we note that the normal BMI group was slightly older than the overweight group, which may reflect population trends in BMI distribution with aging in China. This baseline difference may still represent a potential source of residual confounding. Seventh, although we adjusted the association between calorie intake and mortality for the APACHE II score, AGI grade, and mechanical ventilation, and the results showed consistent trends, we cannot exclude the potential impact of other factors on outcomes. It is unlikely that nutritional intake alone determines prognosis under any circumstance; rather, the observed associations are more plausibly the result of multiple interacting factors, which require further investigation and validation in future studies. Additionally, we only explored subgroup analysis based on the BMI, as we believe that it is closely related to nutritional reserves. Whether other types of critically ill patients also follow the hockey-stick association of calorie delivery still requires further investigation, as this is crucial for individualized nutritional therapy in critically ill patients.

## 5 Conclusion

In our *post-hoc* analysis of a prospective observational study, we observed that the hockey-stick association between daily delivered calories within the first 72 h of ICU admission and 28-day mortality in critically ill patients applies only to those with a BMI of 18.5–24.9 kg/m^2^, whereas no similar hockey-stick association was observed in patients with a BMI of 25–30 kg/m^2^. These findings should be regarded as exploratory and hypothesis-generating, and further studies are needed to confirm and refine these observations.

## Data Availability

The raw data supporting the conclusions of this article will be made available by the authors, without undue reservation.
