# Supplementary material for: The hockey-stick association of energy supply in the first 72 h of critical illness may apply only to patients with a normal body mass index: a post-hoc analysis of a prospective observational multicenter study
Source: Front Nutr. 2026 Jan 9;12:1701067. doi: 10.3389/fnut.2025.1701067 (PMC12827100; doi:10.3389/fnut.2025.1701067)
Supplement: Supplementary file 1 [file Table_1.docx]

**Supplementary document:**

**Hospitals Participating in the Study**

Zhongda Hospital of Southeast University, Nanjing, **East** China

Nanjing General Hospital of Nanjing military Command, Nanjing, **East** China

Zhejiang hospital, Hangzhou, **East** China

First Affiliated Hospital of Sun Yat-sen University, Guangzhou, **South** China

Xiangya Hospital of General south University, Changsha, **Central** China

Zhongnan Hospital of Wuhan University, Wuhan, **Central** China

West China Hospital of Sichuan University, Chengdu, **Southwest** China

First Affiliated Hospital of Xi'an Jiaotong University, Xi’ an, **Northwest** China

First Hospital of Jilin University, Changchun, **Northeast** China

First Hospital of China Medical University, **Northeast** Shenyang, China

First Affiliated Hospital of Dalian Medical University，Dalian, **Northeast** China

Second Affiliated Hospital of Harbin Medical University, Harbin, **Northeast** China


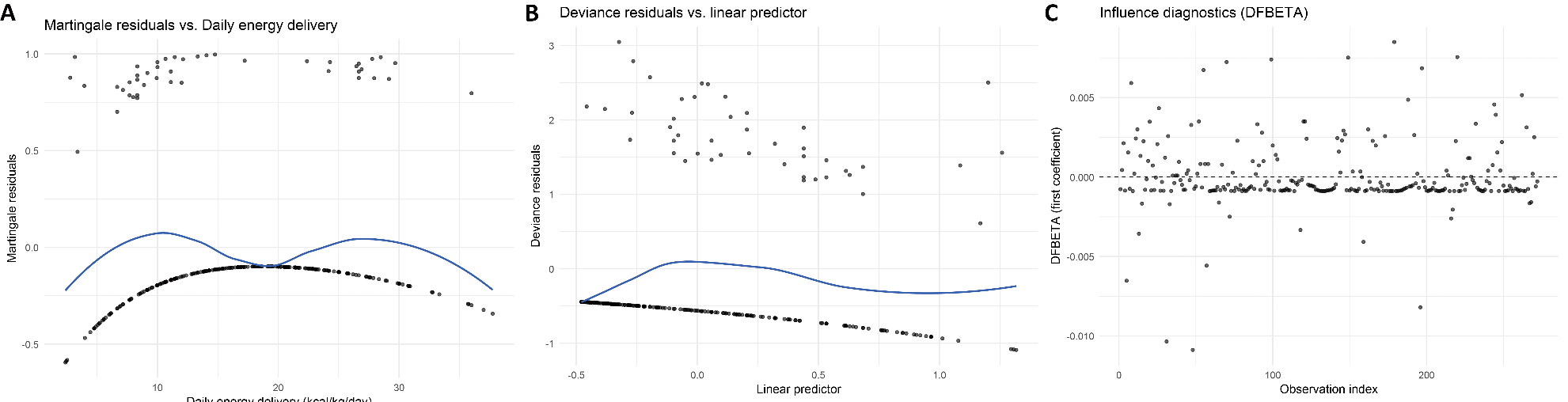


**Figure S1.** Model diagnostics for the Cox model with restricted cubic splines of daily energy delivery in patients with normal BMI.

(A) Martingale residuals versus daily energy delivery with LOESS smooth, showing no strong systematic pattern and suggesting that the spline specification adequately captures the functional form. (B) Deviance residuals versus the linear predictor, without pronounced trends or clustering indicative of major lack of fit. (C) DFBETA values for the spline coefficient, all close to zero, indicating that no single observation exerted undue influence on the model estimates.


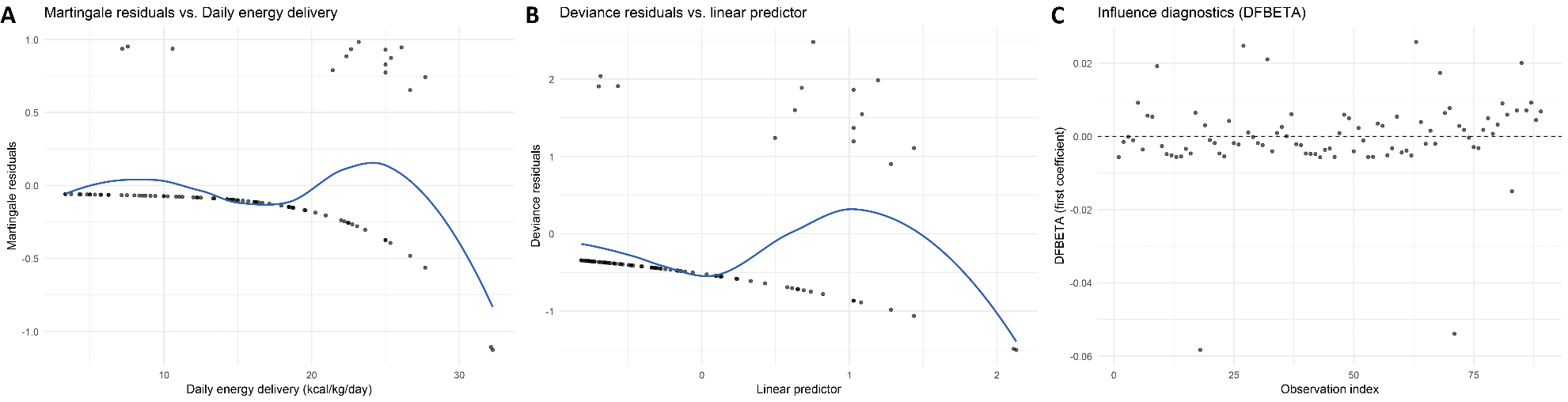
**Figure S2.** Model diagnostics for the Cox model with restricted cubic splines of daily energy delivery in overweight patients.
(A) Martingale residuals versus daily energy delivery with LOESS smooth, showing only mild curvature and no marked systematic deviation from zero. (B) Deviance residuals versus the linear predictor, without clear monotonic trends or dense clusters, suggesting acceptable overall model fit. (C) DFBETA values for the spline coefficient, generally small and well below conventional influence thresholds, indicating absence of highly influential observations.

**Table S1**

The linear association between daily calorie intake and 28-day mortality in the overweight subgroup.

| model | *HR* | (95%*CI)* | *P Value* |
| --- | --- | --- | --- |
| Unadjusted | 1.144 | (1.042 - 1.255) | 0.005 |
| Adjusted (APACHE + AGI) | 1.216 | (1.071 - 1.379) | 0.002 |

Abbreviations: APACHE, Acute Physiology and Chronic Health Evaluation; AGI, acute gastrointestinal injury.
